# Semi-Supervised Adversarial Graph Prediction Model for Industrial Assets

**DOI:** 10.3390/s26196356

**Published:** 2026-10-08

**Authors:** Weijie Kang, Yuze Wei, Junhao Wang, Chaoyin Ma, Bo Liu, Bing He, Guodong Duan

**Affiliations:** 1 Department of Electronic Information Engineering, Rocket Force University of Engineering, Xi’an 710025, China; el1995@stu.xjtu.edu.cn (W.K.); mizi56@126.com (Y.W.); 18781634569@163.com (J.W.); rspaper001@163.com (C.M.); hebing202301@163.com (B.H.); 2 Xi’an Tianhe Defense Technology Co., Ltd., Xi’an 710119, China; thtwliubo@163.com

**Keywords:** adversarial learning, graph neural network, semi-supervised, RUL prediction

## Abstract

**Highlights:**

**What are the main findings?**

- A semi-supervised adversarial graph prediction (SAGP) framework is developed for industrial asset RUL prediction, which unifies graph neural networks, semi-supervised learning, and adversarial training to jointly mine degradation information from labeled full-life failure data and unlabeled incomplete suspension histories.
- A multi-objective loss function with graph adversarial perturbation strategies is designed, and Gaussian distribution-based uncertainty quantification is realized; experiments on tool, aero-engine, and bearing datasets verify that SAGP achieves lower RMSE, MAE, scoring function, and smaller prediction uncertainty than MLP, GraphSage (GSage), and semi-supervised recurrent GAN (SRGAN) under both normal and noisy adversarial conditions.

**What are the implications of the main findings?**

- The graph-based multi-sensor feature modeling scheme explicitly captures the correlation between multi-source monitoring features ignored by traditional Euclidean space methods, providing a graph-based representation for equipment degradation signal analysis under scarce full-life samples.
- The SAGP framework effectively alleviates model overfitting caused by insufficient industrial failure data and enhances prediction robustness against sensor drift and abnormal feature correlations, offering a reliable technical reference for industrial PHM intelligent maintenance and residual life decision-making.

**Abstract:**

In the field of Prognostics and Health Management (PHM), deep learning methods have shown excellent remaining useful life (RUL) prediction ability because of their powerful data fitting ability. Such methods require a large amount of failure history to train, but for industrial assets, their life-cycle data are often insufficient, and effective repairs or replacements are frequently carried out before the failure occurs. On the one hand, these suspension history data cannot be directly used by traditional deep learning methods, resulting in the certain loss of degradation information. On the other hand, the internal structure of industrial assets and the types of features are complex and diverse. Traditional methods use Euclidean space to represent relevant data, ignoring the correlation between features. Therefore, we develop a semi-supervised adversarial graph prediction (SAGP) framework that combines adversarial learning, graph neural networks, and semi-supervised training, using both failure and suspension histories for RUL prediction. Based on graph neural networks, SAGP can effectively obtain the degradation information in the failure and suspension history by representing the degradation data in the form of graphs and setting multi-objective functions. Through semi-supervised learning, SAGP looks for failure and suspension histories similar to the current state and generates simulated data through adversarial training to improve the robustness of the model and finally achieve RUL prediction. Tool, engine, and bearing datasets are used to verify the accuracy and reliability of the SAGP model.

## 1. Introduction

Prognostics and health management (PHM) realizes condition-based maintenance (CBM) through effective condition monitoring and remaining useful life (RUL) prediction of equipment [1]. Accurate RUL prediction can effectively reduce equipment maintenance costs and strengthen safety management, so it has been widely studied and applied in aerospace and other fields [2]. RUL prediction can be achieved using methods such as statistical analysis, knowledge reasoning, data-driven, and even hybrid models [3]. Among them, the data-driven method represented by neural networks has good applicability and prediction accuracy due to its black box processing characteristics and strong data fitting ability [4].

Traditional data-driven methods have achieved good results in the RUL prediction of aero-engines, bearings, and other equipment and can effectively learn the feature characteristics of time series such as vibration and current [5,6]. However, these prediction methods often take each feature value as a parallel input and fail to effectively consider the relationship and influence between each feature [7].

As a new network structure, graph neural network (GNN) can effectively reflect the relationship between each group of data and is suitable for processing non-European spatial data [8]. Nowadays, GNN has developed variants such as graph convolutional neural network (GCN) [9] and graph recurrent neural network (GRNN) [10] and has been used in financial fraud detection [11], recommendation systems [12], anomaly detection [13], etc. In these applications, GNN mainly realizes the functions of node information calculation, data classification, and trend prediction.

**Figure 1:**
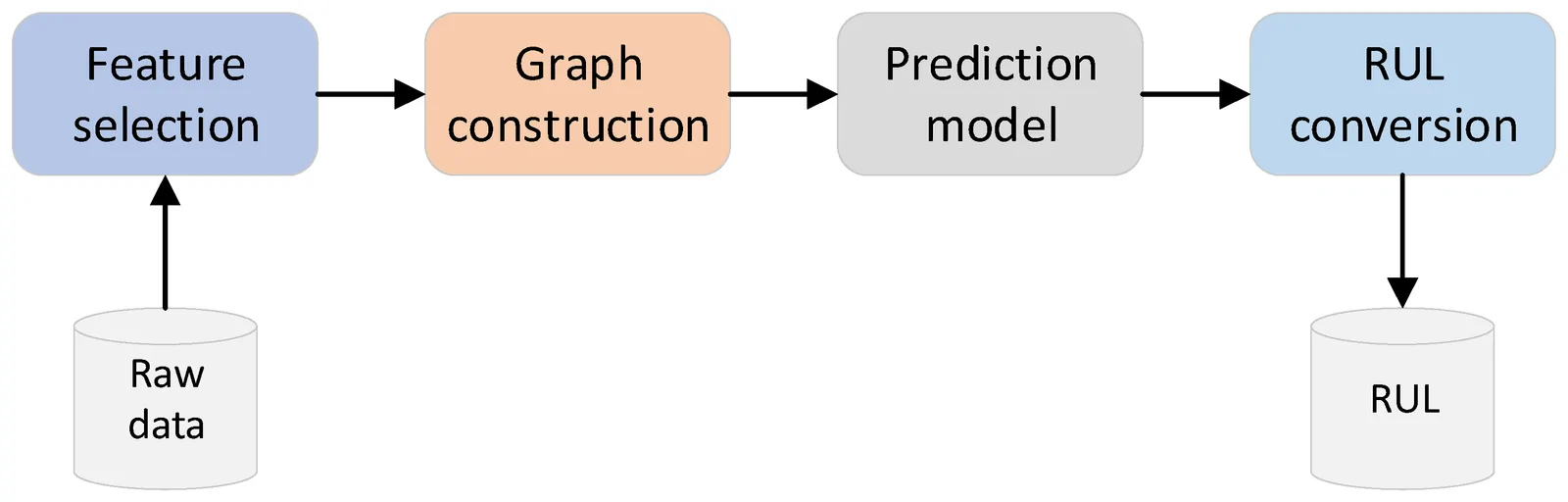
RUL prediction based on graph data.

In recent years, the application of GNN in the field of PHM has also developed. Cui et al. implemented a digital twin-driven graph domain adaptation neural network for RUL prediction of rolling bearings [14]. Zhang et al. proposed a graph neural network-based bearing fault diagnosis method with the Granger causality test [15]. These studies are still limited to fault diagnosis, while Liu et al. completed a graph learning with variational graph structural information bottleneck for multi-sensor machinery remaining useful life prediction [16]. These studies found that GNN can effectively explore the dependencies between multi-sensor data and achieve higher-precision RUL prediction by representing the correlation between multiple features of equipment. Among them, the key to GNN achieving RUL prediction is to use graphs representing the relationship between features. In this type of graph data, nodes represent features, edges represent the relationship between features, and weights reflect the degree of correlation. This graph-based RUL prediction method is shown in Figure 1, and its implementation process is described as feature selection, graph construction, prediction model, and RUL conversion.

The key to achieving accurate and reliable RUL prediction is to obtain sufficient full life-cycle data, but the reality is often that the equipment is repaired before a complete failure occurs, and it is difficult to obtain sufficient failure data [17]. Duan et al. call this incomplete life-cycle data suspension history, and these data also contain rich equipment degradation information [18]. Through semi-supervised learning and other methods, learning the suspension and failure history at the same time can effectively improve the accuracy and reliability of RUL prediction [19]. Therefore, we consider combining GNN with semi-supervised learning and using suspension data to further improve the performance of the prediction model.

However, the scarcity of historical data often exists in industrial assets, which can easily lead to over-fitting of the prediction model [20]. We introduce adversarial learning to improve the robustness and applicability of the prediction model, which also has been applied in the field of health management [21]. Among them, Lei et al. proposed a health status monitoring method based on deep convolutional generative adversarial networks (GAN) under the condition of missing data [22]. Nguyen et al. proposed a physical-infused fuzzy GAN for robust failure prognosis [23]. But these methods all belong to supervised learning, still rely on full life-cycle data, and fail to effectively utilize the suspension data [24]. He et al. proposed a semi-supervised GAN-based RUL prediction model, which can consider failure and suspension histories and effectively improve the prediction accuracy and reliability [25]. However, the basis of this work is still the recurrent neural network, which cannot effectively explore the correlation between features of multi-source data.

On the other hand, semi-supervised adversarial learning has itself become a well-established paradigm, both in its original generative form [26] and on graph-structured data [27,28]; related ideas have also been applied to intelligent fault diagnosis of wind turbines [29], to the design of loss functions for semi-supervised learning [30], and to defect segmentation from images [31]. The present work builds on this line of research and adapts it to the specific requirements of industrial asset RUL prediction.

Building on the generative adversarial learning framework of Goodfellow et al. [26], its extension to graph-structured data [27,28], and recent GNN-based RUL prediction methods [14,15,16], we develop SAGP, a semi-supervised adversarial graph prediction framework adapted to the characteristics of industrial asset RUL prediction. Rather than introducing a fundamentally new adversarial learning paradigm, this work extends the existing paradigm to a setting in which (1) multi-source sensor signals exhibit time-dependent correlations that must be encoded into the graph, and (2) only incomplete suspension histories are available in addition to full-life failure data. The main contributions are as follows:

- A graph-based representation that encodes time-dependent correlations among multi-source sensor signals of industrial assets;
- A semi-supervised adversarial framework that leverages both failure and incomplete suspension histories, extending the semi-supervised graph adversarial paradigm [27,28] to RUL prediction;
- A multi-objective loss that jointly balances prediction accuracy and uncertainty quantification, following the loss-design perspective of [30].

## 2. Overall Technical Scheme

**Figure 2:**
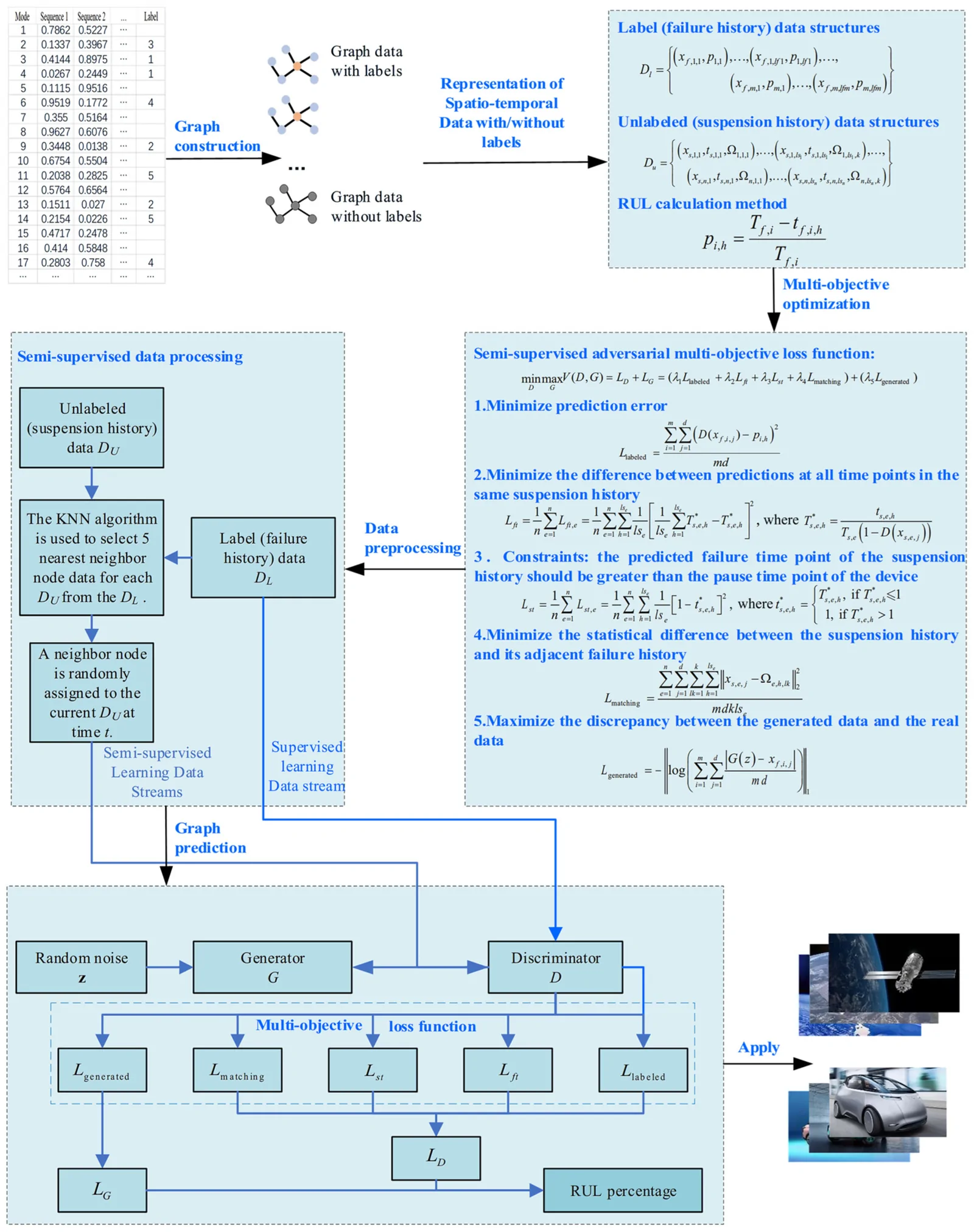
The overall technical scheme of this paper.

For the RUL prediction research of multi-source data, it is necessary to convert different sources into graph data and design a multi-source data representation structure with and without labels. Then, a multi-objective loss function for semi-supervised generation is designed by combining the characteristics of labeled and unlabeled multi-source data. Finally, combining graph structure and semi-supervised adversarial learning, a semi-supervised adversarial RUL prediction method of graph data is designed. The specific idea of this section is shown in Figure 2.

As shown in Figure 2, the scheme comprises three stages. First, multi-source monitoring signals are converted into graph-structured data, in which sensors are represented as nodes and their correlations as edges; both labeled failure histories and unlabeled suspension histories are represented in this form, the latter lacking the end-of-life label. Second, for each unlabeled suspension history, the KNN algorithm selects the five nearest labeled failure histories, and one neighbor node is randomly assigned at each time step so as to build the paired samples required by semi-supervised adversarial training. Third, the generator takes random noise together with the graph embeddings and produces synthetic degradation graphs, while the discriminator distinguishes these synthetic graphs from real ones and simultaneously outputs the RUL percentage; the two networks are optimized jointly under the multi-objective loss defined in Section 3.2, so that both labeled failure histories and unlabeled suspension histories contribute to the prediction.

## 3. Detailed Technical Scheme

### 3.1. Representation of Multi-Source Data with/Without Labels

For clarity, the main symbols used in Equations (1)–(7) are summarized in Table 1.

The health condition data of industrial assets is typically characterized by the coexistence of labeled and unlabeled multi-source data. It is necessary to design a collaborative data representation method to facilitate the application of these labeled and unlabeled multi-source data in the same model. For high-value equipment, it is often repaired before the complete failure of the equipment; it is difficult to obtain enough failure (label) data. Such incomplete life-cycle data is called “suspension history” in the field of health management, which also contains rich equipment degradation information.

In this section, the failure history set is defined as *D_f_* = {*D*_*f*,1_,*D*_*f*,2_,…,*D*_*f*,*m*_}, and the suspension history set as *D_s_* = {*D*_*s*,1_,*D*_*s*,2_,…,*D*_*s*,*n*_}. Here, *m* and *n* denote the number of subsets in the two sets. In general *m* < *n*.

For the fault history subset, take the *i*-th fault history subset *D*_*f*,*i*_ as an example; its representation is composed of monitoring data *x*_*f*,*i*,*j*_ ∈ *R*^1×^*^d^*, graph information {*V*,*E*,*A*} and time nodes *t*_*f*,*i*,*h*_. *f* is the fault history mark; *s* is the suspension history mark; *i* represents the *i*-th fault history, *i* = 1, 2, …, *m*; *j* denotes the *j*-th monitoring data; *d* denotes the number of dimensions; and *h* denotes the *h*-th monitoring time point.

For the suspension history subset, take the *e*-th suspension history subset *D*_*s*,*e*_ as an example; its representation consists of monitoring data *x*_*s*,*e*,*j*_ ∈ *R*^1×^*^d^*, graph information {*V*,*E*,*A*}, and time nodes *t*_*s*,*e*,*h*_. *e* denotes the *e*-th suspension history, *e* = 1, 2, …, *n*; *V* is the set of nodes; *E* is the set of edges; and *A* is the link matrix of the graph. *lf_i_* and *ls_e_* denote the total number of monitoring time points in the *i*-th fault history subset and the *e*-th suspension history subset, respectively. *T*_*f*,*i*_ denote the last time node of the *i*-th fault history subset and the *e*-th suspension history subset, respectively. Taking fault history as an example, when the input is graph data and the output is RUL percentage *p_i, h_*,

(1) $$p_{i,h}=\frac{T_{f,i}-t_{f,i,h}}{T_{f,i}}$$

Therefore, in labeled/unlabeled data representation for industrial assets, the construction of labeled data set based on failure history is described as *D_l_* = {(*x*_*f*,1,1_,*p*_1,1_),…,(*x*_*f*,1,*lf*1_,*p*_1,*lf*1_),…,(*x*_*f*,*m*,1_,*p_m_*_,1_),…,(*x*_*f*,*m*,*lfm*_,*p*_*m*,*lfm*_)}. The lifetime data of the unlabeled (suspension) histories are incomplete and therefore cannot describe the RUL percentage at known time points. We accordingly define it as $D_{u}=\left\{\left(x_{s,1,1},t_{s,1,1},\Omega_{1,1,1}\right),\ldots ,\left(x_{s,1,ls_{1}},t_{s,1,ls_{1}}\right.\right.,\;\left.\left.\Omega_{1,ls_{1},k}\right),\ldots ,\left(x_{s,n,1},t_{s,n,1},\Omega_{n,1,1}\right),\ldots ,\left(x_{s,n,ls_{n}},t_{s,n,ls_{n}},\Omega_{n,ls_{n},k}\right)\right\}$, and Ω_*e*,*h*,*lk*_, *e* = 1,2,…,*n*, *h* = 1,2,…,*ls_e_*, *lk* = 1,2,…,*k* denotes the *lk*-th nearest fault history subset of the *h-*th time point of the *e*-th suspension history subset, which distance is measured by Euclidean distance. A unified graph construction setting is applied to all three datasets (tool, C-MAPSS, and XJTU-SY bearing). Specifically, the KNN algorithm with *k* = 5 nearest neighbors is used; the distance metric is Euclidean; the edge weight is defined as the inverse of the distance $w_{\mathrm{ij}}=\frac{1}{d_{\mathrm{ij}}+\varepsilon }$ with *ε* = 1 × 10^−6^ to avoid division by zero; and the graph is static once constructed and is not updated during training (no edge rewiring across epochs).

### 3.2. Semi-Supervised Adversarial Multi-Objective Loss Function Design

The key to the collaborative application of labeled and unlabeled multi-source data is to design a multi-objective loss function that can simultaneously constrain the labeled and unlabeled data and make full use of the multi-source information in these data. Combining the basic ideas of semi-supervised training and adversarial learning, the multi-objective loss function designed in this section is described as follows:

min*_D_*max*_G_V*(*D*,*G*) = *L_D_* + *L_G_* = (*λ*_1_*L*_labeled_ + *λ*_2_*L_ft_* + *λ*_3_*L_st_* + *λ*_4_*L*_matching_) + (*λ*_5_*L*_generated_) (2)

where *λ_g_*, *g* = 1,2,…,5 is the weight of the corresponding part. The min–max form of *V* (D, G) follows the original generative adversarial framework [26], and the extension of adversarial training to graph-structured data is adopted from [27,28]. *L*_labeled_ is used to minimize the prediction error,

(3) $$L_{\mathrm{labeled}\;}=\frac{\sum_{i=1}^{m}\sum_{j=1}^{d}{\left(D(x_{f,i,j})-p_{i,h}\right)}^{2}}{\mathrm{md}}$$

where *D*(*x*_*f*,*i*,*j*_) represents the output result obtained by inputting *x*_*f*,*i*,*j*_ (the monitoring data of the fault history) into the discriminator, that is, the predicted RUL value, and the meanings of the remaining symbols are consistent with the previous parts.

*L_ft_* is used to minimize the difference between predicted values for all time points in the same suspension history,

(4) $$L_{\mathrm{ft}}=\frac{1}{n}\sum_{e=1}^{n}L_{\mathrm{ft},e}=\frac{1}{n}\sum_{e=1}^{n}\sum_{h=1}^{ls_{e}}\frac{1}{ls_{e}}{\left[\frac{1}{ls_{e}}\sum_{h=1}^{ls_{e}}T_{s,e,h}^{*}-T_{s,e,h}^{*}\right]}^{2},\;\mathrm{with}\;T_{s,e,h}^{*}=\frac{t_{s,e,h}}{T_{s,e}\left(1-D\left(x_{s,e,j}\right)\right)}$$

*L_st_* is a constraint that the predicted failure time point of the suspension history should be greater than the suspension time point of the equipment,

(5) $$L_{\mathrm{st}}=\frac{1}{n}\sum_{e=1}^{n}L_{\mathrm{st},e}=\frac{1}{n}\sum_{e=1}^{n}\sum_{h=1}^{ls_{e}}\frac{1}{ls_{e}}{\left[1-t_{s,e,h}^{*}\right]}^{2},\;\mathrm{with}\;t_{s,e,h}^{*}=\left\{\begin{array}{c} T_{s,e,h}^{*},\;\mathrm{if}\;T_{s,e,h}^{*}⩽1\\ 1,\;\mathrm{if}\;T_{s,e,h}^{*}>1 \end{array}\right.$$

*L*_matching_ is used to minimize the statistical difference between the suspension history and its adjacent failure history,

(6) $$L_{\mathrm{matching}\;}=\frac{\sum_{e=1}^{n}\sum_{j=1}^{d}\sum_{\mathrm{lk}=1}^{k}\sum_{h=1}^{ls_{e}}{\left\|x_{s,e,j}-\Omega_{e,h,\mathrm{lk}}\right\|}_{2}^{2}}{\mathrm{mdkl}s_{e}}$$

*L*_generated_ is used to maximize the difference between the generated data and the real data,

(7) $$L_{\mathrm{generated}\;}=-{\left\|\log\left(\sum_{i=1}^{m}\sum_{j=1}^{d}\frac{\left|G\left(z\right)-x_{f,i,j}\right|}{\mathrm{md}}\right)\right\|}_{1}$$

where ‖‖_1_ and ‖‖_2_ denote the first-order and second-order norm, respectively. The formulation of the adversarial loss follows [30], where discriminative adversarial networks are used to learn the loss for semi-supervised learning. Optimization objective: The model is trained under the standard GAN min–max objective min*_D_*max*_G_V*(*D*,*G*) defined in Equation (2). Training sequence: In each iteration, the discriminator is updated once first, and then the generator is updated once; this 1-to-1 update ratio is held throughout training. with an SGD with momentum of 0.9 and an initial learning rate of 0.01. The learning rate decays by 90% every 1500 iterations, up to a maximum of 6000 iterations, with a batch size of 16; *λ*_1_, …, *λ*_5_ are initialized to 0.2 and adjusted during training by monitoring the gradient balance (see Section 4 for the adjustment schedule).

Unlike traditional domain adaptation models (e.g., semi-supervised recurrent GAN) that rely on recurrent neural networks (RNN) for sequential data modeling, SAGP leverages GNN to capture spatial-temporal dependencies among sensor features dynamically. For instance, while semi-supervised recurrent GAN aligns feature distributions via adversarial training in Euclidean space, SAGP integrates adversarial perturbations directly into the graph structure (e.g., edge weight variations and node noise), enabling robust handling of sensor drift and cross-condition degradation. Compared to the representative GNN model GSage, which focuses solely on feature aggregation via neighborhood sampling, SAGP’s semi-supervised adversarial framework jointly optimizes labeled failure data and unlabeled suspension histories, reducing overfitting in data-scarce scenarios. Additionally, unlike classical feedforward non-graph networks and multi-layer perceptron (MLP)-based approaches that treat sensor measurements as independent inputs, SAGP explicitly models feature correlations through graph edges, enhancing interpretability and prediction stability.

### 3.3. Semi-Supervised Adversarial RUL Prediction in Graph Data

**Figure 3:**
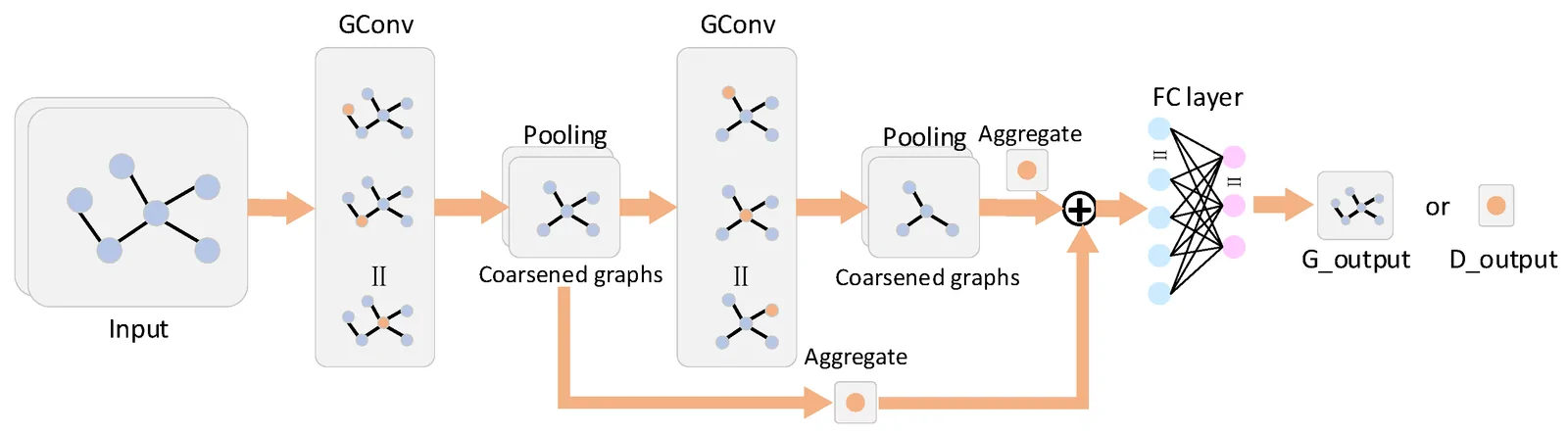
GNN-based generator and discriminator in GAN.

Except for operations such as graph construction, pooling, and aggregation of data, GNNs are basically the same as ordinary neural networks. Combined with the idea of adversarial training, the GNN-based generator and discriminator in GAN are represented by Figure 3.

First, the data are constructed graphically, and then two convolution and pooling operations are performed in sequence. The results obtained from the two pooling operations are then aggregated and are finally output as graphic data or a single value through the fully connected (FC) layer. Among them, the graphic data corresponds to the generated graphic data output by the generator in the GAN, and the single value corresponds to the discrimination result output by the discriminator. We note that multi-generator and multi-discriminator architectures have also been explored in adversarial learning [31]; here, a single generator and a single discriminator are adopted, since the prediction task requires a single degradation representation, and the dual-network design already provides sufficient capacity while keeping the training cost tractable.

**Figure 4:**
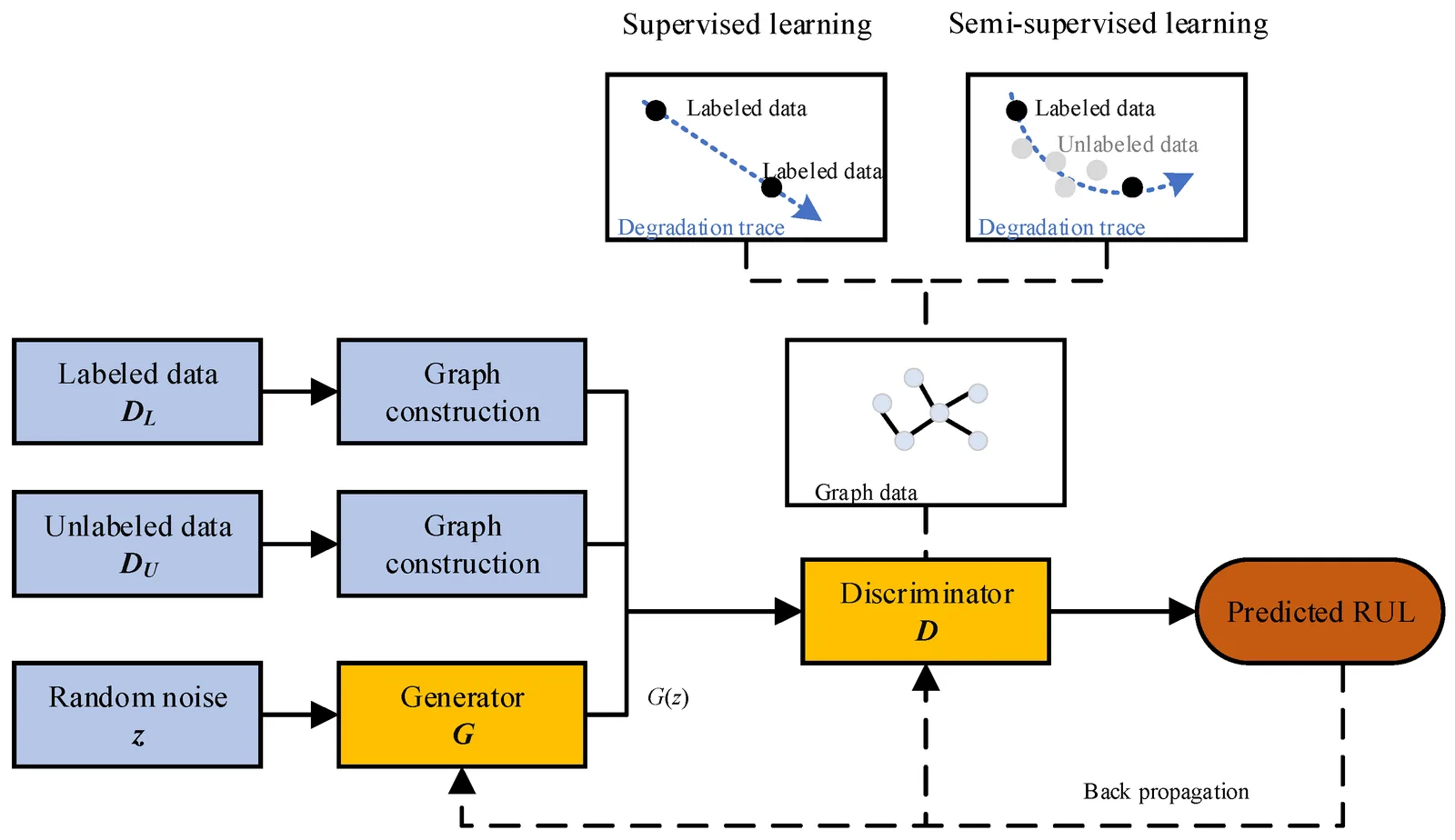
The implementation process of SAGP.

Therefore, the implementation process of SAGP is described as Figure 4.

Figure 4 illustrates the key differences between SAGP and baseline models. While semi-supervised GAN uses a generator-discriminator pair to synthesize sequential data, SAGP generates synthetic degradation graphs with perturbed node features and edges. GSage, though graph-based, lacks adversarial training and semi-supervised constraints, limiting its ability to generalize across operational conditions. MLP, as a non-graph method, fails to exploit spatial dependencies.

The SAGP workflow comprises three stages:

1. Graph Construction: Sensor data are converted into graph nodes (features) and edges (feature correlations via KNN with *k* = 5; Euclidean distance; inverse-distance edge weight; the graph is static after construction).
2. Adversarial Training:
  - Generator (G): Receives 200D noise and graph embeddings to generate synthetic degradation graphs;
  - Discriminator (D): Classifies real vs. synthetic graphs and predicts RUL;
3. Multi-objective Optimization: The losses *L*_labeled_, *L_st_*, *L*_matching_ are weighted by *λ_g_* and optimized via SGD with momentum (0.9). Hyperparameters (e.g., initial learning rate 0.01, dropout 0.2) were selected via a grid search on a validation subset (20% of the FD001 training data). The weights *λ_g_* were dynamically adjusted during training by monitoring gradient balance.

During training, the generator and discriminator losses were monitored to maintain a balance between the two networks, and the loss weights *λ_k_* were adjusted accordingly. A dedicated visualization of the adversarial training process, such as those proposed in [32,33], is a promising direction for future work.

For the collaborative application of labeled/unlabeled multi-source data, not only the loss function should be considered, but also the model structure and process should be designed. In this section, when the semi-supervised adversarial graph RUL prediction is carried out, firstly, the KNN (K nearest neighbors) algorithm is used on the suspension history data to select a number of nearest neighbor node data for each suspension history from the failure history. Secondly, a neighbor node was randomly assigned for the paused historical data of the current input prediction model at time t. Finally, combined with the multi-objective loss function, the RUL prediction based on the generative graph data was carried out.

In the SAGP framework, adversarial attacks are simulated through two primary perturbations:

1. Node Feature Noise Injection: The generator introduces Gaussian noise to sensor measurements (node features) to mimic sensor drift. For example, in the CMAPSS dataset, adversarial examples are generated by perturbing sensor values as *x_adv_* = *x* + *N*(0,0.1), where *x* denotes raw sensor data.
2. Edge Weight Manipulation: The correlation strength between features (edge weights in the graph) is randomly altered by ±10% to simulate abnormal sensor interactions. This disrupts the spatial dependencies learned by the GNN. The discriminator is trained to distinguish these adversarial graphs from real data via the loss *L*_generated_, which penalizes synthetic perturbations (Equation (7)). Simultaneously, the generator iteratively refines its perturbations to bypass the discriminator, leading to a robustness equilibrium.

Attack target, timing, and range: Both perturbations act on the input graph, i.e., on the node feature matrix *x* and on the edge weights; the RUL labels are never modified. The perturbation is applied in two steps: (1) each node feature is corrupted by adding zero-mean Gaussian noise with standard deviation 0.1, *x_adv_* = *x* + ε with ε ~ *N*(0, 0.1^2^); and (2) each edge weight is scaled by a factor (1 + δ), where δ is drawn independently from the uniform distribution U(−0.1, 0.1). During training, these perturbations are produced by the generator as part of the min–max game with the discriminator. At evaluation time (Section 5), an independent perturbation with exactly the same settings is applied to the test graphs of all compared methods. The magnitudes are kept identical for every method under comparison.

The perturbation procedure is summarized in Algorithm 1.
**Algorithm 1:** Adversarial perturbation used in SAGP**Input:** graph *G* = (*x*, *A*) with node features *x* and edge weights *A*;            noise scale *σ* = 0.1; edge perturbation ratio *r* = 0.1.**Output:** perturbed graph *Ḡ* = (*x_adv_*,*A_adv_*).1**Step 1:** Node feature perturbation
2              *x_adv_* ← *x* + *ε*,             *ε* ~ N(0, σ^2^) *node feature noise injection*3**Step 2:** Edge weight perturbation
4              *A_adv_* ← *A*
5             **for** each edge (*i*, *j*) of *A* **do**
6                    *δ_ij_* ~ U (−*r*, *r*)*edge weight manipulation*7                    *A_adv_*[*i*, j] ← *A*[*i*, j] · (1 + *δ_ij_*)
8             **end for**

9**Step 3:** Output the perturbed graph
10             **return** *Ḡ* = (*x_adv_*,*A_adv_*)


In order to realize the RUL prediction of multi-source information fusion, this section firstly introduces the failure history, suspension history, and labeled/unlabeled data and designs a labeled/unlabeled multi-source data representation structure. Secondly, based on the characteristics of semi-supervised training and generative models, a multi-objective loss function is designed. Finally, the implementation process of semi-supervised adversarial graph RUL prediction is described.

## 4. Model Analysis of Outputs and Parameters

In RUL prediction research, effective quantitative analysis of the uncertainty contained in the prediction results is crucial, especially when the amount of data is small. The uncertainty of the prediction result is mainly composed of the noise contained in the input data and the error generated by the prediction model. Among them, since the input data generally contains noise with unknown distribution, these input data are often assumed to obey a Gaussian distribution with a mean *μ*_*i*,*j*_ and a variance $\sigma_{i,j}^{2}$. The conjugate prior distribution of the Gaussian distribution is described as follows [34]:

(8) $$p_{i,j}\sim N\left(\mu_{i,j},\sigma_{i,j}^{2}\right),\;\mathrm{with}\;\mu_{i,j}\sim N\left(\gamma_{i,j},\sigma_{i,j}^{2}\nu_{i,j}^{-1}\right),\sigma_{i,j}^{2}\sim \Gamma^{-1}\left(\alpha_{i,j},\beta_{i,j}\right)$$

where Γ() denotes the Gamma distribution, *γ*_*i*,*j*_ is the mean of *μ*_*i*,*j*_, $\sigma_{i,j}^{2}\nu_{i,j}^{-1}$ is the variance of $\sigma_{i,j}^{2}$, and *α*_*i*,*j*_ and *β*_*i*,*j*_ are the relevant parameters of the Gamma distribution. Therefore, the output value of the prediction model is described as a Gaussian distribution with mean E(*μ*) and variance E(*σ*^2^).

(9) $$E(\mu )=\hat{\gamma },E\left(\sigma^{2}\right)=\frac{\hat{\beta }}{\hat{\alpha }-1}$$

Therefore, the value of *σ*^2^ describes the noise contained in the input data of the prediction model, and the variance of *μ* describes the uncertainty of the prediction result [35]. According to the related research in [36], when the prediction model is constructed from precise data, the variance of the mean of the prediction results should fluctuate within a small range. Therefore, we can use the variance of the mean of the prediction results to measure the uncertainty contained in the prediction model. The literature [25] proposes a credit value (CV) to evaluate the reliability of the output results of a prediction model.

(10) $$\mathrm{CV}=\sum_{i=1}^{n_{t}}\mathrm{Var}\left(\mu_{i}\right)=\sum_{i=1}^{n_{t}}\frac{\beta_{i}}{\nu_{i}\left(\alpha_{i}-1\right)}$$

Among them, *n_t_* represents all test data. The value of CV can effectively reflect the uncertainty of the model. When the CV is small, the confidence of the predicted RUL is higher. Therefore, the uncertainty of the prediction results can be evaluated during the operation of the GAN model.

In addition, since the prediction result conforms to the Gaussian distribution, the confidence interval of the prediction result can also be obtained by calculation. The confidence interval for the 95% confidence level of the prediction result is as follows:

(11) $$\left[E(\mu )-2\sqrt{E\left(\sigma^{2}\right)},E(\mu )+2\sqrt{E\left(\sigma^{2}\right)}\right]$$

For GAN, the training process of the model requires special attention, which involves the setting and optimization of parameters. In the SAGP framework, the discriminator and the generator are trained with a 1-to-1 update ratio (see Section 3.2): in each iteration, the discriminator is updated first, and then the generator is updated once. The objective function min*_D_*max*_G_V*(*D*,*G*) = *L_D_* + *L_G_* = (*λ*_1_*L*_labeled_ + *λ*_2_*L_ft_* + *λ*_3_*L_st_* + *λ*_4_*L*_matching_) + (*λ*_5_*L*_generated_) of the SAGP contains multiple subtasks, and *λ_i_*, *i* = 1,2,…,5 is the weight corresponding to each subtask.

Among them, *λ*_1_ represents the weight of the label data, that is, the proportion of the failure history data in the prediction model during the supervised learning process. In the model training process, the same learning weight (*λ_i_* = 0.2, *i* = 1,2,…,5) is first set so that the loss function values of each part have similar sizes. Second, the value of *λ*_1_ is adjusted multiple times during training, and the one *λ*_1_^*^ that minimizes the value of the objective function is determined. Finally, *λ*_1_^*^ and *λ_i_* = 0.2, *i* = 2,3,4,5 are used to retrain the model.

## 5. Case Study

### 5.1. Experimental Background

This section uses three examples to validate the SAGP model.

The first part uses PHM 2010 data [37]. This data set is the wear state monitoring (*c*_1_,*c*_2_,⋯,*c*_6_) of the tool life cycle of the high-speed CNC machine tool, which is repeated six times under specific conditions. The hosting links on the original competition page are no longer functional; the dataset can be obtained from the mirror given in the Data Availability Statement. The dataset partitioning follows three distinct categories: (1) labeled failure histories comprising complete life-cycle data from trials *c*_1_,*c*_4_,*c*_6_; (2) unlabeled suspension histories consisting of 50% samples from trials *c*_2_,*c*_3_,*c*_5_, and (3) a test set consisting of the remaining 50% of those trials. In this setting, the same tool appears in both training and evaluation, but at strictly disjoint time intervals, and no RUL label of any test segment is used during training. This structured division enables rigorous validation of model generalizability across both labeled and unlabeled degradation patterns.

The detection values of seven different sensors are recorded in each set of data, namely the cutting force and vibration signals in the X, Y and Z directions, and the effective value (AE-RMS) of the platform sound signal. The settings in reference [38] are used to convert tool wear predictions into RUL predictions. This transformation is described as follows:

RUL(*t*) = *t_f_* − *t* (12)

where *t* represents the current time, RUL(*t*) represents the RUL of the current time, and *t_f_* represents the time when the tool reaches the fault threshold. Referring to related research, the threshold is generally set to 0.16 mm.

The sensor monitoring values in this dataset also need to be normalized so that their values could fall between [0, 1]. The processing method is as follows.

(13) $${\hat{y}}_{i}=\frac{y_{i}-\min(y)}{\max(y)-\min(y)}$$

During the training process of the prediction model, all the data in *c*_1_,*c*_4_,*c*_6_ are used as the labeled failure history data, while for *c*_2_,*c*_3_,*c*_5_, the early portion of each monitoring sequence is used as the unlabeled suspension history and the remaining samples from the test set.

The second part is to use CMAPSS data [39]. This dataset includes four sets of data, of which FD001 contains one operating condition and one failure mode, FD002 contains six operating conditions and one failure mode, FD003 contains one operating condition and two failure modes, and FD004 contains six operating conditions and two failure modes. Each set of data includes a training set and a test set. The training set contains the full life-cycle data, while the test set only contains part of the initial-stage data. The standard RUL of each engine is defined as 125 flight cycles. Each group of data records 24 kinds of monitoring data in each flight cycle, of which the first three are working condition data, and the rest are sensor monitoring values. However, some of these data do not change or fluctuate irregularly, which should be eliminated during the experiment. The data of different working conditions are clustered by the k-means method and normalized for all data. Each group of data is trained separately. The dataset partitioning strategy for the CMAPSS benchmark is structured as follows: (1) labeled failure histories comprising the complete training sets from FD001 to FD004, which include full life-cycle degradation profiles; (2) unlabeled suspension histories obtained by truncating the early portion of each test unit at equidistant temporal intervals; and (3) a test set consisting of the later portion of the same units. In this setting, the same device appears in both training and evaluation, but at strictly disjoint time intervals; no RUL label of any test segment is used during training.

On the nature of this setting: since the suspension histories come from the test data, the training procedure sees the input distribution of the test data, although no test RUL label is used at any stage; the setting is therefore transductive rather than purely inductive. This is deliberate, as it matches the practical PHM scenario, where monitoring data from operating assets are available before their end-of-life labels. We nevertheless note it as a limitation: the results should be interpreted under a transductive (within-device) protocol, and a strictly inductive evaluation is left for future work.

The third part extends the validation of the proposed SAGP model to the XJTU-SY bearing dataset, a publicly available benchmark for RUL prediction under accelerated life testing conditions. The dataset comprises run-to-failure vibration signals from 15 rolling element bearings under three distinct operational conditions (35 Hz, 37.5 Hz, and 40 Hz). Each bearing’s vibration data is sampled at 25.6 kHz in horizontal and vertical directions, with explicit annotations of failure modes (e.g., outer race cracks, inner race wear, cage fracture). The dataset’s multi-failure characteristics make it ideal for validating the robustness and generalizability of SAGP. The data segmentation is described as (1) labeled failure data, full life-cycle data from bearings 2_2, 2_4, and 2_5; (2) unlabeled suspension data, the first 230 time points of bearings 2_1 and 2_3, selected via stratified random sampling to cover early degradation stages; and (3) test set, the remaining portions of bearings 2_1 (261 samples) and 2_3 (303 samples), i.e., all time points recorded after the first 230. The same two bearings thus contribute to both training and evaluation, but at strictly disjoint time intervals, and no RUL label of the test segments is used during training. This within-device protocol is consistent with that described above for CMAPSS.

The relevant parameters of the network are the generator and discriminator of GAN, which respectively contain two double-layer graph convolution parts, and the number of units in each layer of the convolution part is 1024. There is also a double-layer fully connected layer; the number of units in the first layer is 1024, the number of units in the second layer of the generator is determined by experimental data, and the number of units in the second layer of the discriminator is 1. The input to the generator is 200-dimensional random noise, and the input to the discriminator is determined by experimental data. Both mean aggregation and EdgePool operations are performed in the network. The remaining parameters include the initial learning rate of 0.01, the dropout ratio of 0.2, and the activation function using ReLU. The momentum parameter is 0.9 and is optimized using static gradient descent (SGD). The learning rate decays by 90% every 1500 iterations, and the maximum number of iterations is 6000. The number of neighbors selected for each time node in the suspension history is set to 5. Each group of experiments is repeated 10 times, and the average value is taken as the final experimental result. For each method, the relative change with respect to its clean-data value is reported together with the corresponding results, which permits a direct comparison of robustness across methods. It should be noted that the 10 repetitions were performed to obtain stable average values, and the per-run results were not retained separately; consequently, a per-run standard deviation and a paired significance test are not reported here, and a full statistical evaluation of robustness is left for future work. In addition, all experiments were accomplished with Anaconda and Python 3.8 and executed on a computer with an Intel Core i7-1165G7 (2.80 GHz) CPU, 16 GB memory, and a Windows 10 Enterprise operating system.

For effective comparison, the typical method GSage [40] in GNN, SRGAN [41] in semi-supervised GAN, and the common method MLP [42] are selected for comparative experiments. All methods are trained and evaluated on exactly the same data partition and on the same test units. SRGAN is semi-supervised and is therefore given the same unlabeled suspension histories as SAGP. MLP and GSage are fully supervised and cannot exploit unlabeled data; they are trained on the labeled failure histories only and are reported as supervised reference baselines rather than as strictly matched semi-supervised competitors. To verify that the advantage of SAGP does not arise solely from the additional unlabeled data, we note that SRGAN, which is given exactly the same unlabeled suspension histories as SAGP, is consistently outperformed by SAGP on all three datasets. A full ablation of SAGP trained without suspension histories is left for future work.

The prediction results are evaluated using the root mean squared error (RMSE) and the score function (SF). RMSE and SF are defined as follows:

(14) $$\mathrm{RMSE}=\sqrt{\frac{1}{n_{t}}\sum_{i=1}^{n_{t}}{\left(\delta_{i}\right)}^{2}}$$

(15) $$\mathrm{SF}=\sum_{i=1}^{n_{t}}SF_{i},\;\;\;\;\mathrm{with}\;\;\;SF_{i}=\left\{\begin{array}{cc} e^{-\frac{\delta_{i}}{\frac{13}{3}}-1,} & \delta_{i}<0\\ e^{\frac{\delta_{i}}{33}}-1, & \delta_{i}⩾0 \end{array}\right.$$

where SF is the evaluation score, *δ_i_* = *RUL*_ture_ − *RUL_predicted_*.

### 5.2. Case Study 1: Tool Dataset

**Figure 5:**
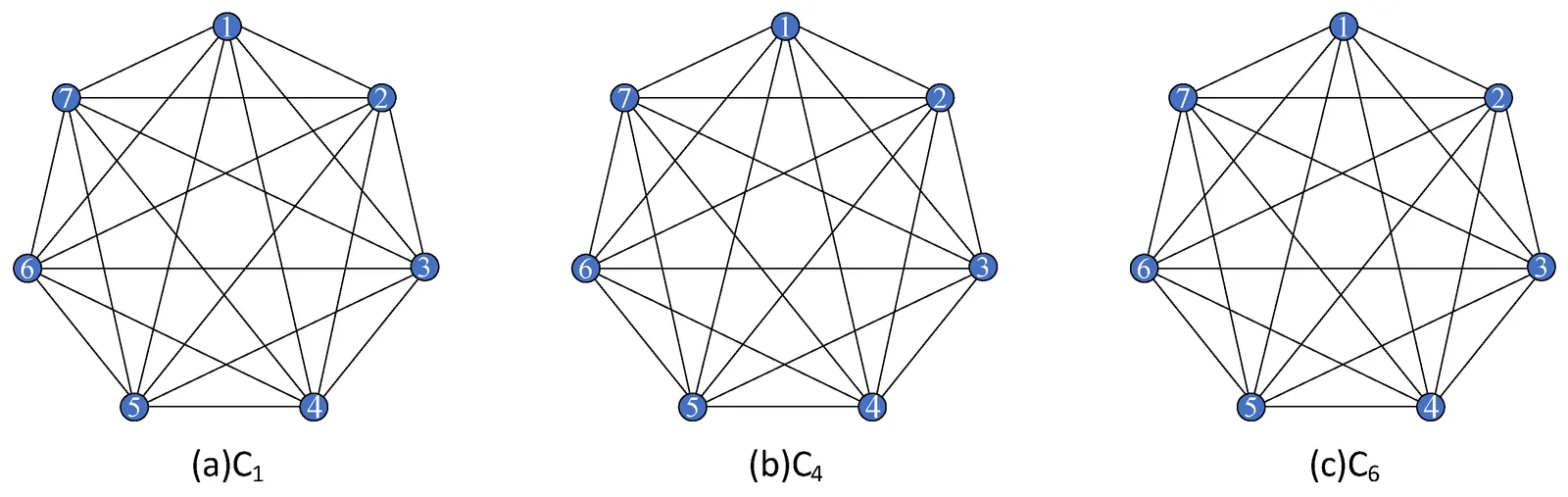
The sensor’s graph structure of the PHM2010 dataset.

For the 7-dimensional data in the PHM2010 dataset, the basic sensor map structure is constructed as shown in Figure 5.

Nodes 1 to 7 correspond to the cutting force and vibration signals in the X, Y, and Z directions, respectively, as well as the effective value (AE-RMS) of the platform sound signal.

**Figure 6:**
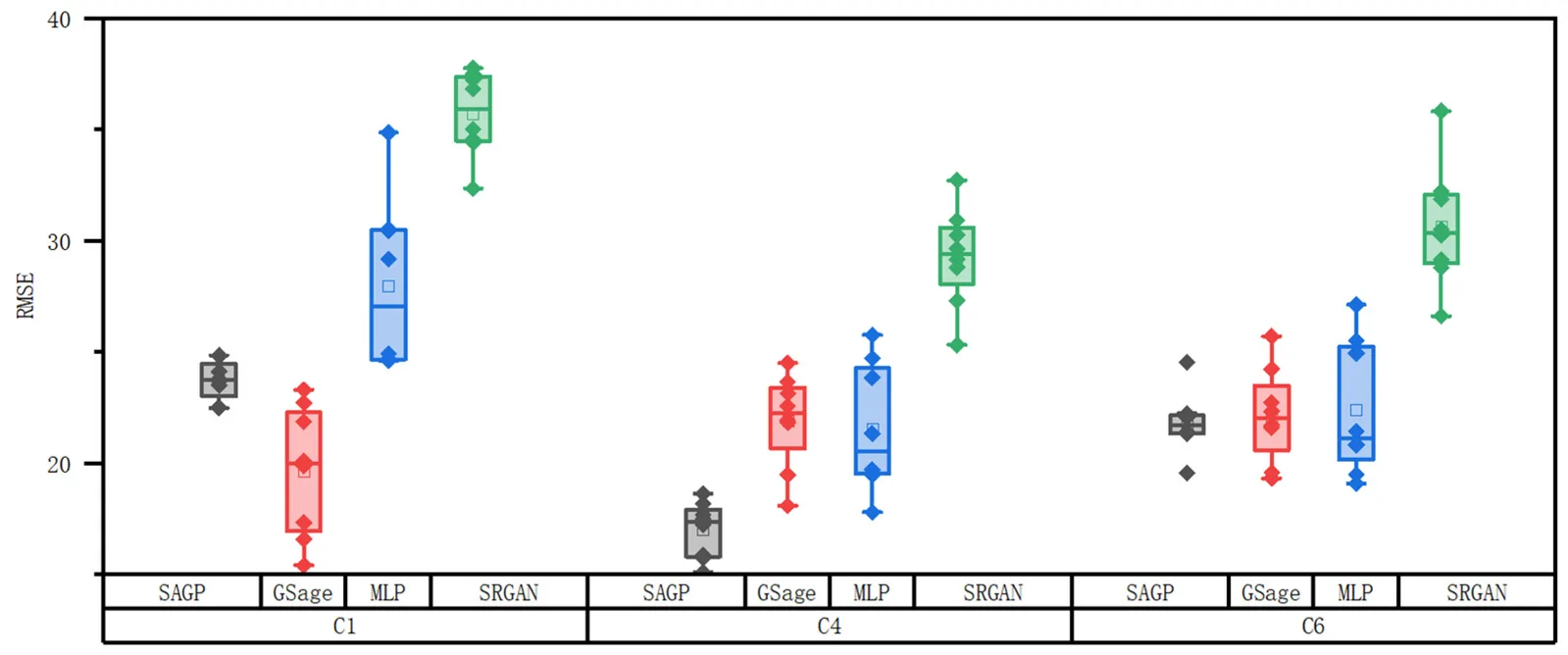
Prediction results (RMSE values) of SAGP, GSage, MLP, and SRGAN on the PHM2010 dataset.

RUL predictions are performed on this dataset using GSage, SRGAN, MLP, and SAGP, and box plots are used to describe the experimental results. The lower edge of the box is the 25% quantile (Q1), the horizontal line in the box is the median, and the upper edge of the box is the 75% quantile (Q3). □ is the average value of the data, and ◇ is the observed value of each run. The interquartile range (IQR) is defined as IQR = Q3 − Q1. The lower whisker is the smallest observed value (Q1 − 1.5 IQR), and the upper whisker is the largest observed value (Q3 + 1.5 IQR). The RMSE results obtained by running each method 10 times are shown in Figure 6.

From the experimental results, it is found that the RMSE values of each method are not very different, but the prediction accuracy of SRGAN is lower than that of the other three methods. The performance of the GSage method is generally close to that of the MLP method, and the average performance of the GSage method is the best among the three comparison methods in the C1 dataset. However, the SAGP model has higher prediction accuracy in each dataset, and the prediction stability is always higher than other methods. This shows that under the semi-supervised learning architecture, by introducing a graph structure and adversarial training, the stability of the prediction method is effectively improved.

**Figure 7:**
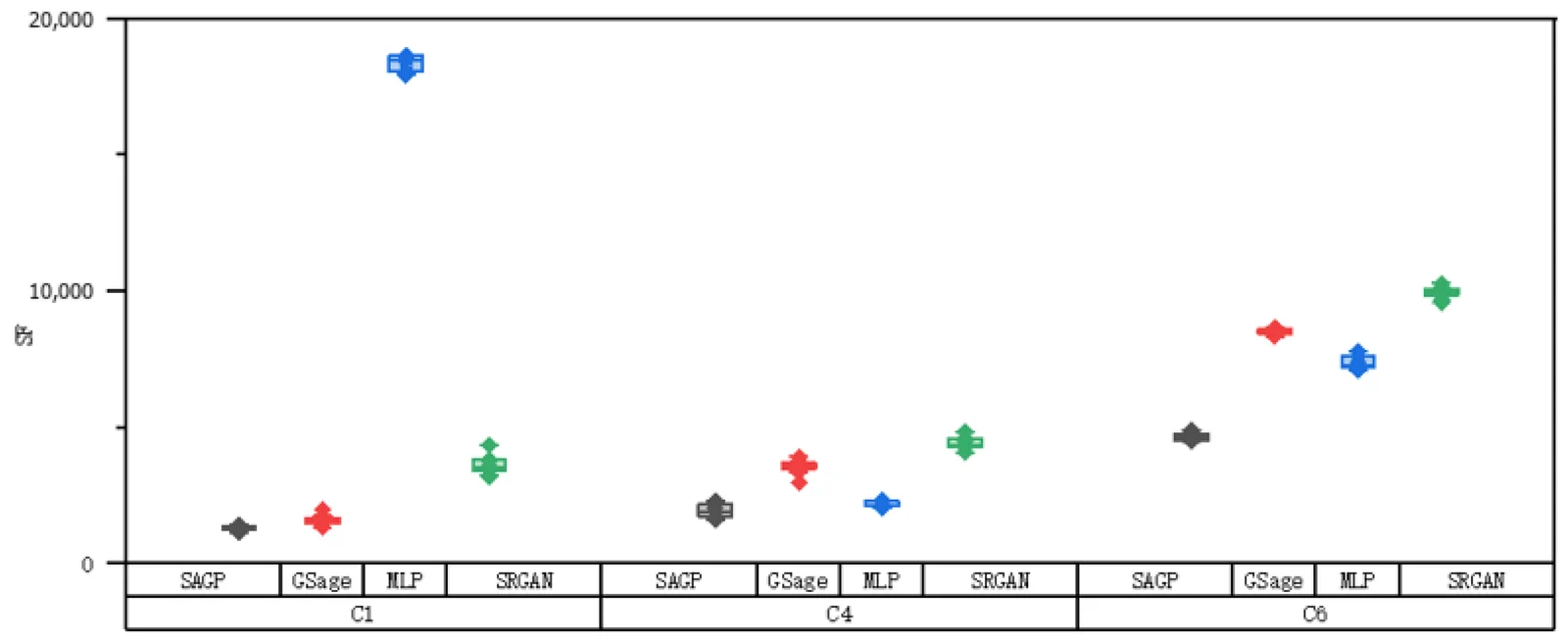
Predicted SF values of MLP, SRGAN, GSage, and SAGP on the PHM2010 dataset.

Next, the SF values obtained by running each method 10 times are shown in Figure 7.

The experimental results demonstrate that the SF values of each method are quite different. The MLP method deviates significantly on the C_1_ dataset, which confirms the significant fluctuation of the RMSE of this method on the C_1_ dataset in Figure 6. The GSage method performs well on the C_1_ dataset but underperforms on the C_4_ and C_6_ datasets. The SRGAN method performs poorly on each dataset, which is consistent with the results of the RMSE. Overall, the SAGP model has shown good prediction accuracy in each dataset, and the prediction stability is also outstanding.

**Figure 8:**
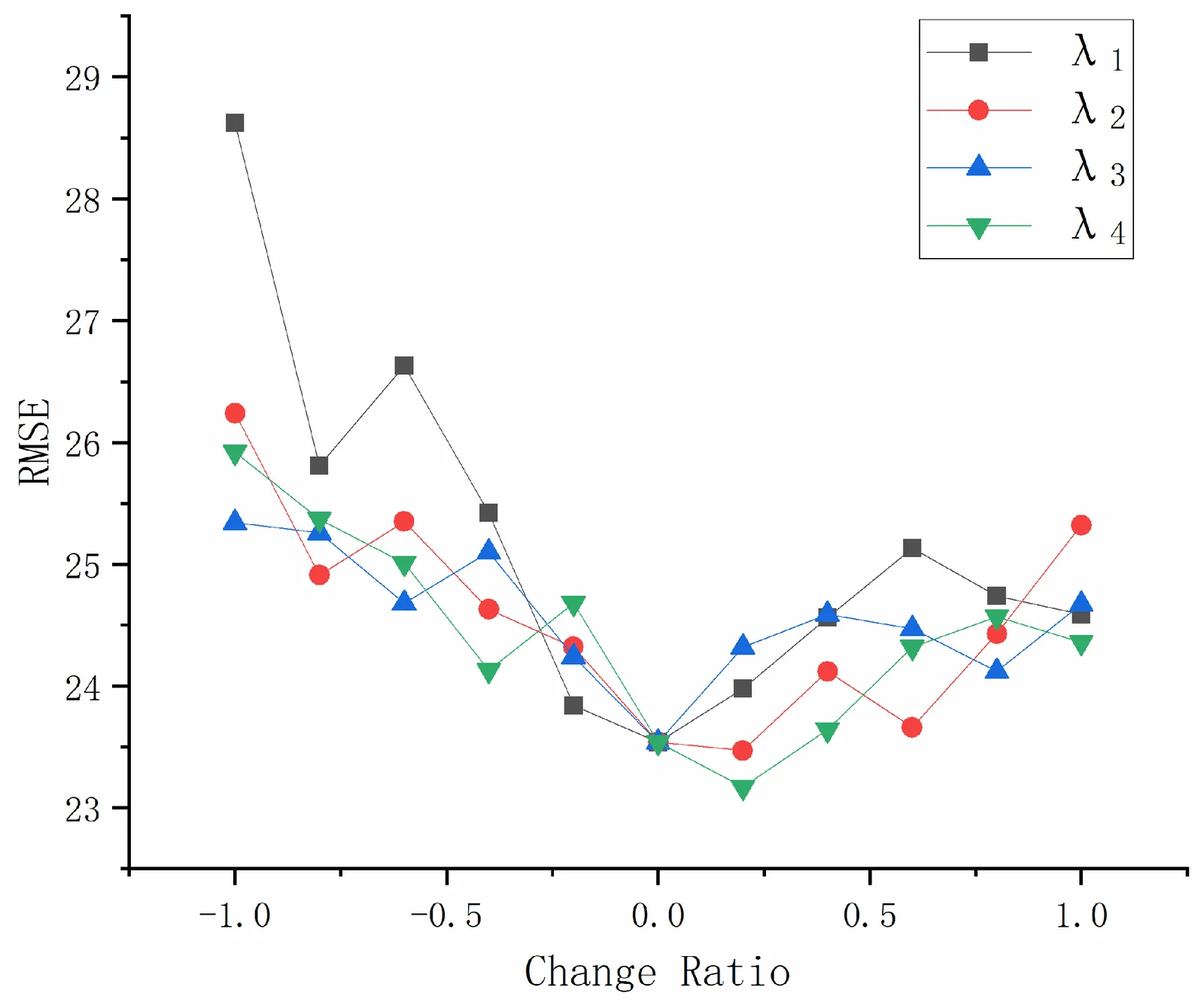
The effect of parameter changes in the SAGP model on the RMSE value of the prediction results.

Finally, the influence of parameters *λ_i_*, *i* = 1,2,…,5 in the semi-supervised model on the RUL prediction results is analyzed. Since *λ*_5_ is mainly related to the generator, the rest of the parameters are related to the discriminator. Therefore, on the basis of fixed *λ*_5_, the remaining *λ_i_* are changed, and the fluctuation of the RUL prediction result is observed. Since *λ*_1_^*^ must be obtained by multiple trainings, its value may be quite different from *λ_i_*, *i* = 2,3,4. Therefore, by increasing or decreasing the value of each *λ_i_* by a relative percentage (20%), the unification of the parameter change amount is achieved. Based on the C_1_ data set, the related experiments were repeated 10 times, and the average results were obtained as shown in Figure 8.

The experimental results demonstrate that the parameters *λ*_1_ have a relatively large influence on the RUL prediction results. This is due to the fact that the failure history contains richer equipment health information, and when the value of *λ*_1_ is decreased, the RUL prediction results drop rapidly. But it is worth noting that the change of a single parameter has no obvious effect on the RUL prediction results. For example, when the values of *λ*_2_, *λ*_3_, and *λ*_4_ change, the overall fluctuation of the RMSE value is small, the maximum impact is only 11.5%, and the rest are all within 10%. The related research of the SRGAN model found that when the relevant parameters of the model are set properly, the change of a certain weight parameter in the objective function will not have a significant impact on the prediction results [25]. The experiment proves that the prediction model based on SAGP also satisfies this rule. In this section, SAGP uses both the failure history and the suspension history to construct the prediction model, which improves the stability of the prediction results.

### 5.3. Case Study 2: Aero-Engine Dataset

**Figure 9:**
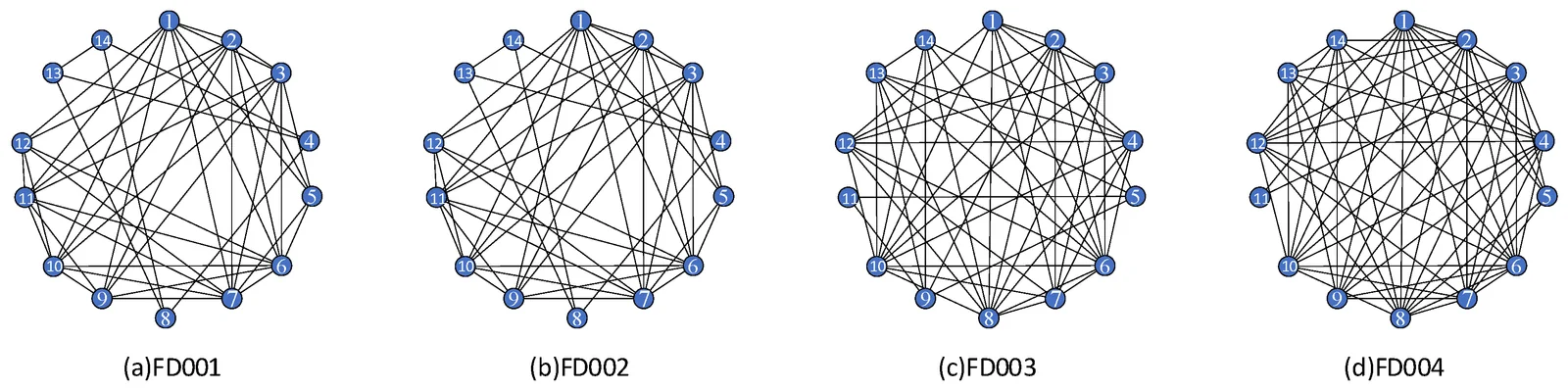
The sensor’s graph structure of the CMAPSS dataset.

Since the monitoring values of some sensors in the CMAPSS data are always unchanged, only sensors 2, 3, 4, 7, 8, 9, 11, 12, 13, 14, 15, 17, 20, and 21 are selected as the model’s input volume [43]. The sensor graph composed of these 14-dimensional data is constructed using the KNN method (k = 5) [16], and its structure is shown in Figure 9.

Among them, nodes 1 to 14 are sorted and distributed according to the value of the sensor.

**Figure 10:**
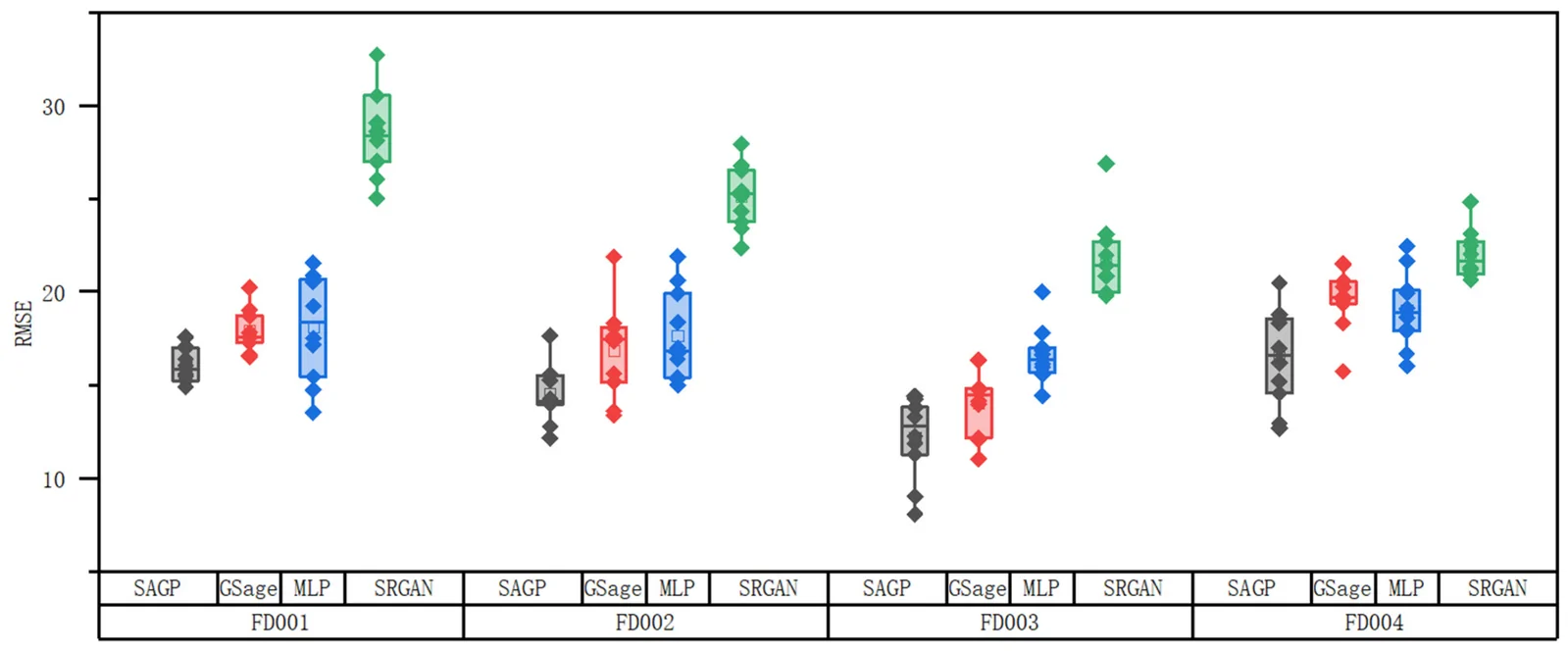
Prediction results: RMSE values of MLP, SRGAN, GSage, and SAGP on the CMAPSS dataset.

The RMSE results obtained by running each method 10 times are shown in Figure 10.

The above experimental results presented above demonstrate that the prediction accuracy of the SRGAN method is not as good as that of other methods on each dataset. The RMSE values of GSage and MLP on different datasets have their own advantages, and the fluctuation of GSage is relatively smaller. The SAGP model has the smallest average RMSE value on the four data sets, and the fluctuations are small except for FD004.

**Figure 11:**
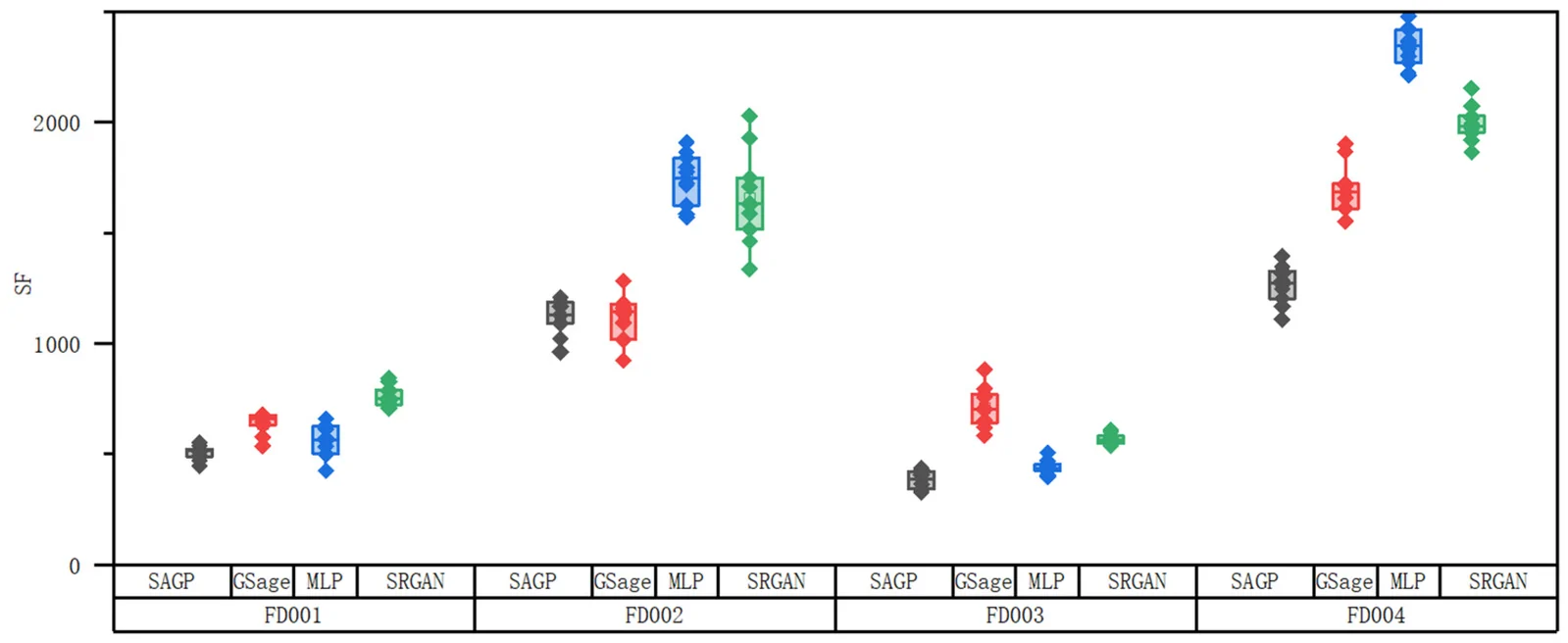
Predicted SF values of MLP, SRGAN, GSage, and SAGP on the CMAPSS dataset.

Next, each method was run 10 times, and the SF results obtained are shown in Figure 11.

The experimental results show that the SF values of FD001 and FD003 are generally smaller than those of FD002 and FD004, which indicates that the complexity of the working conditions will affect the prediction accuracy. For the FD001 dataset, each method has good prediction accuracy. Relatively speaking, the SAGP model has the highest accuracy and the smallest fluctuation. For the FD002 dataset, the prediction accuracy of SAGP and GSage is higher, but the relative fluctuation of SAGP is smaller. For the FD003 dataset, SAGP has the highest prediction accuracy, but MLP shows extremely high stability and relatively high prediction accuracy. For the FD004 dataset, SAGP has the highest prediction accuracy and is close to other methods in terms of stability. Overall, the SAGP model has good prediction accuracy and stability on each dataset.

**Figure 12:**
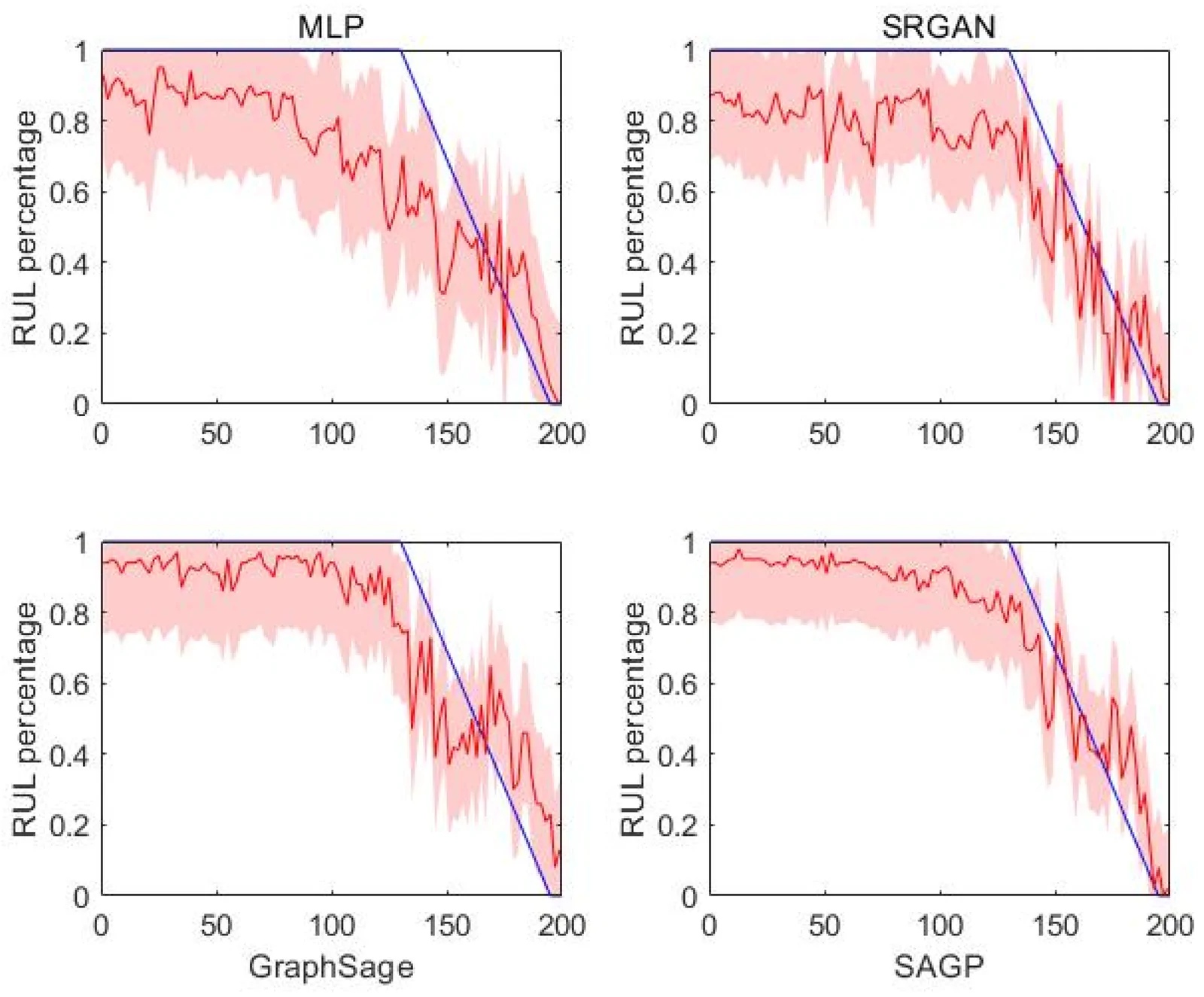
Prediction results of MLP, SRGAN, GSage, and SAGP in the third group of data in the FD001 test set.

Finally, the uncertainty contained in the prediction results of each model is analyzed. Taking the third group of data in the FD001 test set as an example, CV is used to measure the uncertainty in the prediction results of each method. According to the previous correlation analysis, the predicted RUL values of each method are assumed to follow a normal distribution *N* (*μ*, *σ*^2^). The prediction results of each method are shown in Figure 12.

In Figure 12, the blue line represents the true RUL value, the red line represents the mean of the predicted results, and the light red area represents the 95% confidence interval of the predicted results. The experimental results show that the MLP method fails to accurately predict the degradation trend of this set of data, and the premature aging of prediction results appears. SRGAN can better track the degradation trend in the degradation stage, but there are obvious fluctuations in the entire life cycle. The GSage method has relatively stable prediction results in the initial healthy stage of the equipment but cannot accurately predict its degradation trend in the degradation stage. Although the confidence intervals of the prediction results of each method can roughly reflect the aging trend of the equipment, the prediction results of SAGP are closest to the real RUL, and the confidence interval is relatively narrower, so it has higher prediction accuracy and reliability.

**Figure 13:**
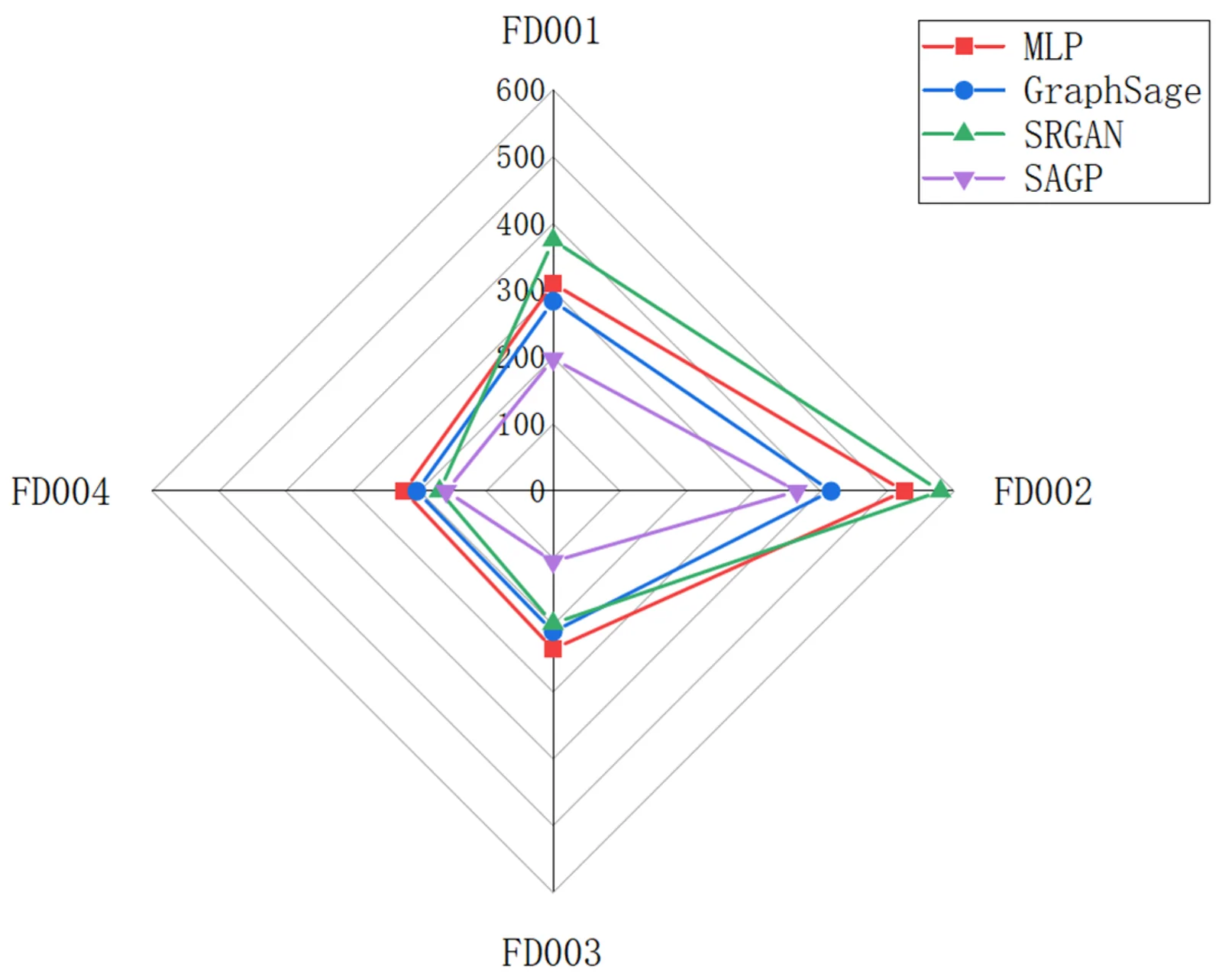
The average CV value of the prediction results of MLP, SRGAN, GSage, and SAGP on the CMAPSS dataset.

The above experiment only evaluates the operating state of a single piece of equipment as an example. In order to evaluate the uncertainty of the prediction methods in a wider range, predictions were performed on each data set, and the average CV value was calculated. The results are shown in Figure 13.

From the above results, it was determined that the average CV value for FD002 in each prediction method is the highest, and the average CV value for FD004 is the lowest, which corresponds to the RMSE value distribution of the prediction results in Figure 10. Specifically, for the FD001 and FD002 datasets, the average CV values of each method show a significant difference; the MLP method has the largest uncertainty, while the SAGP model has the smallest uncertainty. For the FD003 dataset, the average CV values of MLP, GSage, and SRGAN are relatively close, which are all larger than those of the SAGP model. For the FD004 dataset, the average CV values of each method are relatively close. Overall, the performance of each method varies on different datasets, but SAGP shows high prediction reliability on all datasets, which is also consistent with the previous experimental results of a single engine life cycle.

### 5.4. Case Study 3: Bearing Dataset

**Figure 14:**
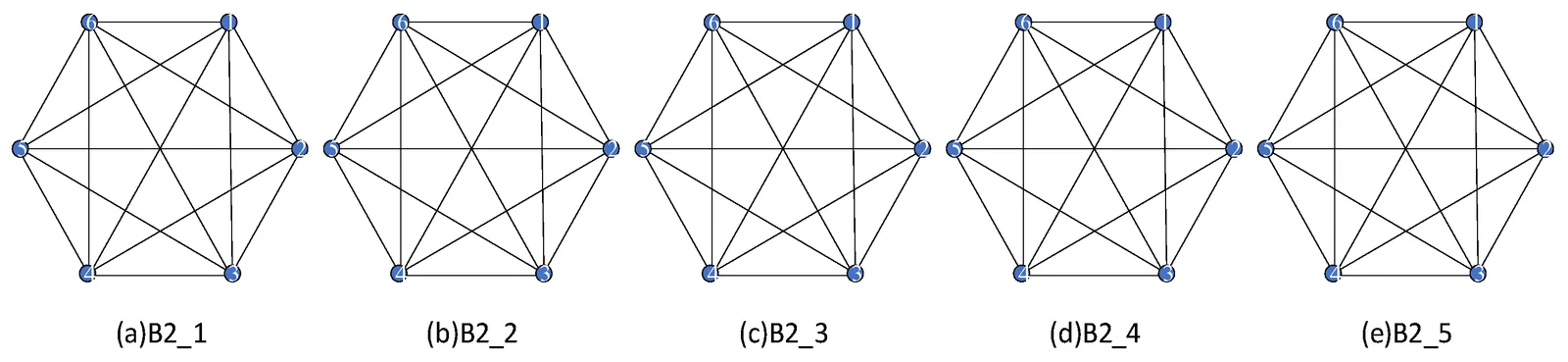
The sensor’s graph structure of the XJTU-SY dataset.

The XJTU-SY dataset has only horizontal and vertical vibration signals, so it is not suitable to use a graph structure to represent the spatial relationship between multi-dimensional features. However, in the actual data process, signal processing methods are usually used to extract features from the time domain, frequency domain, and time-frequency domain, respectively, to reflect the degradation process of bearings from normal state to severe failure. Therefore, based on the RMS value in the time domain, the average amplitude in the frequency domain, and the wavelet packet band energy ratio in the horizontal and vertical directions [44], this section constructs the basic graph structure as shown in Figure 14.

Nodes 1 to 6 are the time domain RMS, the frequency domain average amplitude, and the wavelet packet band energy ratio in the horizontal and vertical directions, respectively.

The two previous experiments use RMSE, SF, and CV values to evaluate the prediction accuracy, but RMSE is sensitive to outliers because it magnifies large errors by squaring, while SF and CV place greater emphasis on the volatility of the prediction results. In this section, we consider the MAE (mean absolute error) metric to evaluate the prediction accuracy of the algorithm. This index is more stable and robust to noise interference and is suitable for scenarios that need to balance the influence of outliers; especially when the noise of industrial data is large, it has more reference value. The calculation method is as in (16).

(16) $$\mathrm{MAE}=\frac{1}{N}\sum_{i=1}^{N}\left|\mathrm{RU}L_{\mathrm{ture}\;}-\mathrm{RU}L_{\mathrm{predicted}}\right|$$

**Figure 15:**
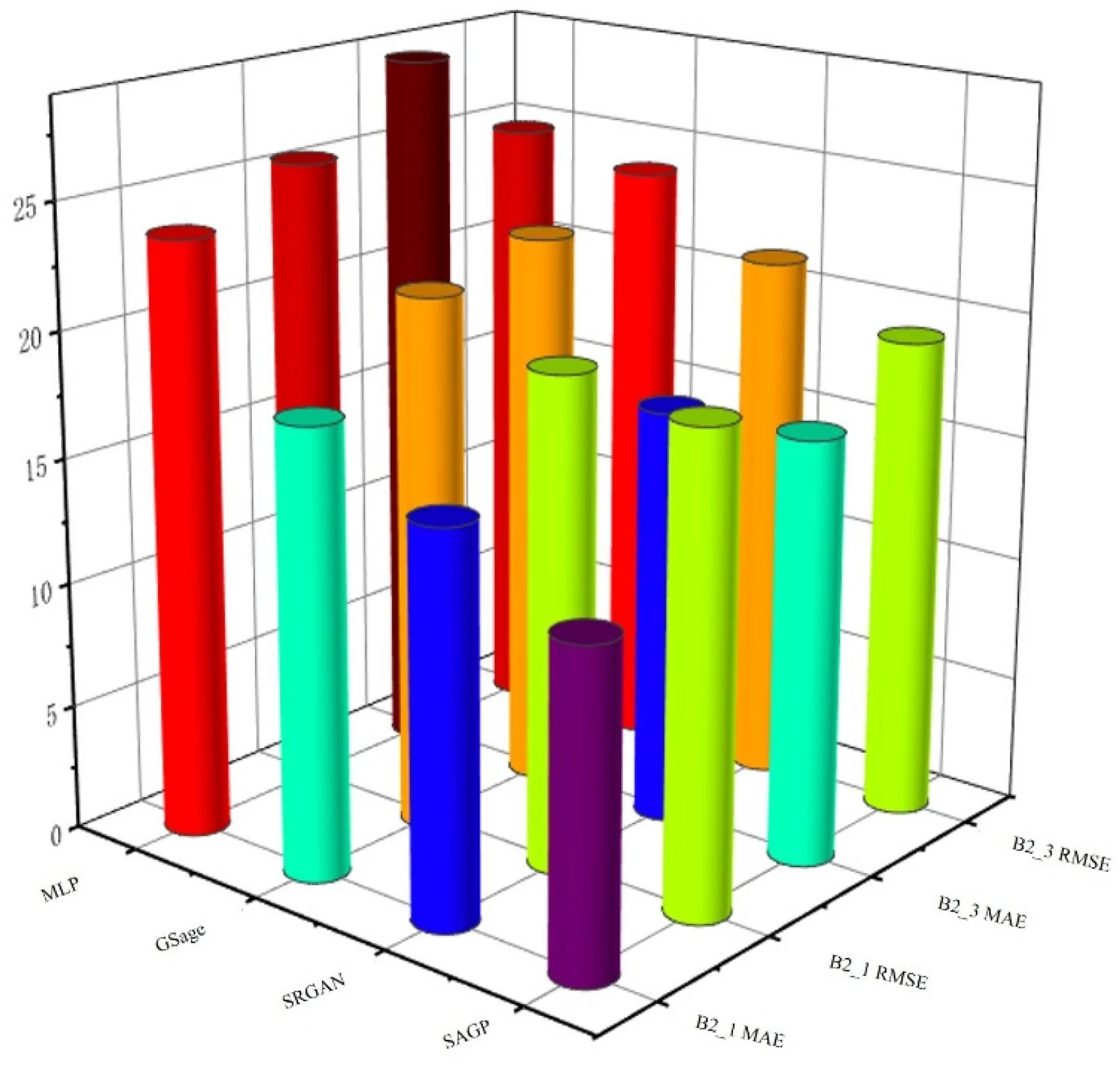
The average MAE and RMSE values of the prediction results of MLP, SRGAN, GSage, and SAGP on the XJTU-SY dataset.

The average MAE and RMSE results obtained by running each method 10 times are shown in Figure 15.

The experimental results show that the SAGP method has higher prediction accuracy than other methods. The SAGP method integrates time domain, frequency domain, and time-frequency domain features through hexagonal graphs to enhance the degradation characterization ability and then jointly optimizes labeled and unlabeled data. The SAGP method performs best under the background of two sets of test data belonging to different fault types (outer ring crack and cage fracture), which verifies the versatility of the model.

To further quantify the robustness of the SAGP method, we inject adversarial noise into the B2_1 test data using exactly the same perturbation settings as in training (Gaussian node noise with σ = 0.1 and ±10% edge weight perturbation). The same perturbed test graphs are fed to every compared method so that all methods are evaluated under identical data visibility. The average results of 10 repeated experiments are shown in Table 2. A systematic sensitivity study over a wider range of perturbation strengths is left for future work.

Under adversarial conditions, SAGP exhibited the smallest degradation in prediction accuracy. Its MAE increased by only 9.67% (from 12.73 to 13.96), significantly lower than MLP (+18.93%), SRGAN (+14.04%), and GSage (+13.12%). Similarly, its RMSE rose by only 6.08% (from 18.91 to 20.06), outperforming MLP (+16.49%) and SRGAN (+10.62%). This highlights SAGP’s resilience to adversarial perturbations, attributed to its semi-supervised adversarial training framework, which dynamically balances synthetic and real data during optimization. Dynamic noise injection simulates a complex industrial scenario to verify the robustness of the proposed method.

The SAGP framework demonstrates distinct superiority over traditional domain adaptation models by dynamically modeling spatiotemporal dependencies among sensor features through graph structures, which are often overlooked in feature-alignment-based approaches. Unlike domain adaptation methods that statically align cross-domain distributions, SAGP integrates adversarial learning with graph neural networks to synthesize degradation patterns under environmental perturbations (e.g., ±10% edge weight variations and Gaussian noise injection). This semi-supervised adversarial mechanism enables robust handling of domain shifts while preserving intrinsic sensor correlations. Additionally, SAGP attains comparable accuracy using only 50% of unlabeled data, highlighting its data efficiency. These advancements position SAGP as a versatile solution for industrial scenarios requiring both precision and adaptability to heterogeneous operational environments.

The computational demands of SAGP were evaluated on an Intel Core i7-1165G7 CPU (16 GB RAM), requiring 4.2 h (6000 iterations) for training with a peak memory usage of 8 GB. While this exceeds baseline methods (MLP: 0.8 h, GSage: 1.5 h), the overhead arises from dual-network adversarial training and graph operations (edge pooling, neighborhood aggregation). However, in industrial deployments, GPU acceleration and sparse matrix optimizations can effectively reduce training time. For mission-critical applications (e.g., aerospace), SAGP’s superior accuracy (RMSE value in the experiment) and reliability (CV value in the experiment) justify its resource cost. As industrial computing infrastructure advances, the practical impact of computational overhead diminishes, while prediction precision remains paramount for safety and maintenance optimization.

In this section, three examples are used to verify the prediction accuracy and reliability of the SAGP model. The SAGP model combines the information of failure and suspension historical data and introduces the GNN model under the framework of semi-supervised learning. Through the adversarial training process, the prediction accuracy and reliability of the model are further improved.

## 6. Conclusions

With the development of PHM technology, signal relationship, random disturbance, suspension history, etc., need to be taken into consideration. In this paper, the semi-supervised learning model is introduced into the historical data of suspension combined with the historical data of failures to effectively enrich the degradation information of the prediction model. By using adversarial training, the generated data with random disturbance effectively improves the robustness of the prediction model. By using the GNN model, the spatial relationship between the internal features of the industrial assets is effectively sorted out, and the accuracy of the prediction model is further improved. By combining semi-supervised learning, adversarial training, and graph neural networks, we develop the SAGP framework, which adapts the semi-supervised adversarial learning paradigm to industrial asset RUL prediction. Compared with MLP, GSage, and SRGAN, the prediction accuracy is significantly improved.

This work is a first exploration of semi-supervised adversarial GNN RUL prediction, a direction that is still at an early stage. Future work will focus on two extensions: adaptive and more computationally efficient graph construction, for example, dynamic graph updating instead of the static graph adopted here, and a systematic study of how the weights of the individual sub-losses in the multi-objective function affect the generated data so as to obtain more targeted RUL prediction for graph data.

## Figures and Tables

**Table 1:** Notation used in Equations (1)–(7).

Symbol	Description
*D*_*f*,*i*_/*D*_*s*,*e*_	*i*-th labeled failure history/*e*-th unlabeled suspension history
*m*/*n*	number of failure histories/number of suspension histories
*x*_*f*,*i*,*j*_	*j*-th monitoring sample of the *i*-th failure history
*d*	number of monitoring dimensions
*T*_*f*,*i*_	total time length of the *i*-th failure history
*t*_*f*,*i*,*h*_	time index of the *h*-th sampling point
*p*_*i*,*h*_	RUL percentage at the *h*-th time point
*ls_e_*	number of time points of the *e*-th suspension history
$T_{s,e,h}^{*}/t_{s,e,h}^{*}$	time stamp after KNN-based imputation/after constraint
Ω_*e*,*h*,*lk*_	*lk*-th nearest neighbor of the *e*-th suspension history at time *h*
*k*	number of nearest neighbors (*k* = 5)
*V*/*E*/*A*	node set/edge set/adjacency matrix of the graph
*G*(·)/*D*(·)	generator network/discriminator network
*z*	noise vector (200 dimensions)
*λ_k_*(*k* = 1,…,5)	weight of the *k*-th sub-loss

**Table 2:** Robustness comparison of MAE and RMSE under adversarial attacks.

Method	Clean MAE	Adversarial MAE	Clean RMSE	Adversarial RMSE
MLP	23.72	28.21 (+18.93%)	25.53	29.74 (+16.49%)
GSage	17.84	20.18 (+13.12%)	21.35	22.90 (+7.26%)
SRGAN	15.46	17.63 (+14.04%)	19.58	21.66 (+10.62%)
SAGP	12.73	13.96 (+9.67%)	18.91	20.06 (+6.08%)

## Data Availability

The data used in this paper are publicly available. The PHM2010 tool-wear dataset is the 2010 PHM Society Conference Data Challenge dataset [37]. The file-hosting links on the official competition page are no longer functional; the dataset is therefore available from the mirror at https://www.kaggle.com/datasets/rabahba/phm-data-challenge-2010 (accessed on 18 September 2026). The C-MAPSS dataset [39] is available from the NASA Prognostics Data Repository at https://www.nasa.gov/intelligent-systems-division/discovery-and-systems-health/pcoe/pcoe-data-set-repository/ (accessed on 18 September 2026). The XJTU-SY bearing dataset [44] is available at https://biaowang.tech/xjtu-sy-bearing-datasets (accessed on 18 September 2026).
